# Confounders of Ultrasound Attenuation Imaging in a Linear Probe Using the Canon Aplio i800 System: A Phantom Study

**DOI:** 10.3390/diagnostics14030271

**Published:** 2024-01-26

**Authors:** Olivia Hänni, Lisa Ruby, Catherine Paverd, Thomas Frauenfelder, Marga B. Rominger, Alexander Martin

**Affiliations:** 1 Faculty of Medicine, University of Zurich, Dekanat Pestalozzistrasse 3, 8032 Zurich, Switzerland; 2 Institute of Diagnostic and Interventional Radiology, University Hospital Zurich, Rämistrasse 100, 8091 Zurich, Switzerlandmarga.rominger@usz.ch (M.B.R.); 3 Department of Radiology, Memorial Sloan Kettering Cancer Center, 1275 York Avenue, New York, NY 10065, USA

**Keywords:** ultrasound, attenuation, fat, liver, phantom

## Abstract

There have been studies showing attenuation imaging (ATI) with ultrasound as an approach to diagnose liver diseases such as steatosis or cirrhosis. So far, this technique has only been used on a convex probe. The goal of the study was to investigate the feasibility of ATI measurements using the linear array on a canon Aplio i800 scanner on certified phantoms. Three certified liver tissue attenuation phantoms were measured in five different positions using a linear probe. The effects of positioning and depth were explored and compared. The values were compared to the certified expected value for each phantom as well as the different measurement values for each measurement position. The ATI measurements on phantoms showed significant effect for the different probe positions and region of interest (ROI) depths. Values taken in the center with the probe perpendicular to the phantom were closest to certified values. Median values at 2.5–4.5 cm depth for phantoms 1 and 2 and 0.5–2.5 cm for phantom 3 were comparable with certified values. Measurements taken at a depth greater than 6 cm in any position were the least representative of the certified values (*p*-value < 0.01) and had the widest range throughout the different sessions. ATI measurements can be performed with the linear probe in phantoms; however, careful consideration should be given to depth dependency, as it can significantly affect measurement values. Remaining measurements at various depths within the 0.5–6.0 cm range showed deviation from the certified values of approximately 25%.

## 1. Introduction

Non-alcoholic hepatic steatosis is a condition present in 20–30% of the general population in the Western world, which is characterized by the accumulation of triacylglycerol (TAG)-rich fat droplets within hepatocytes [1,2]. Non-alcoholic fatty liver disease (NAFLD) is now the most important cause of chronic liver disease worldwide, ranging from simple steatosis to steatohepatitis (NASH), fibrosis, cirrhosis and hepatocellular carcinoma [1,3].

Studies have shown that the ultrasound attenuation imaging (ATI) with a convex array is an accurate diagnostic method for tissue changes in liver, especially steatosis [4,5,6,7,8], but the same technique has yet to be fully explored using a linear probe. Attenuation imaging has been used by Canon in the Aplio series of devices to determining the attenuation coefficient in the assessment of non-alcoholic fatty liver disease [9,10,11]. A range of clinical attenuation cut-off values have been published for hepatic steatosis [6,8,9,10,11,12,13,14,15,16]. Previously published values using the Canon Aplio i800 system and convex probe range from >0.59–0.69 for a steatosis score of ≥S1 and >0.65–0.72 dB/cm/MHz for a steatosis score of ≥S2 [5,9,10,11,12,13].

High attenuation of ultrasound signals leads to poor image quality at depth, especially when evaluating deep abdominal organs. Ultrasound signal attenuation is dependent on physical properties of the tissue being measured and the depth of the measurement [17]. For example, when imaging fatty liver, there is an associated increase in ultrasound signal attenuation due to an increase in backscatter [18], as well as absorption [19]. This increase in attenuation causes a decrease in signal intensity, leading to poor image quality in deeper regions. 

The advantages of using ultrasound as a screening method or tool to diagnose and monitor diseases are well known and widely discussed, such as the non-invasive nature, the accessibility and availability of equipment, the low costs, and the painless nature of the procedure [16,20,21,22]. Furthermore, no additional equipment is needed for examinations with ATI [23] and it has been shown to be more sensitive than controlled attenuation parameter (CAP, “FibroScan”, Echosens, Paris, France), which is clinically used for screenings and diagnostics in liver diseases [13,21] to detect moderate to severe steatosis of the liver [23].

Being able to measure the attenuation coefficient in tissue for ultrasound on a linear probe would allow the early detection of changes in more superficial tissues, such as breast tissue [24] (determining breast tissue density and lesions), muscles [25] (sarcopenia) or the thyroid [26] (nodule assessment), or it may also allow for the evaluation of more superficial liver lesions. For example, Qin et al. have shown that the high-frequency linear ultrasound probe was more sensitive in detecting superficial lesions than the convex probe and suggest combining both in screening the liver [27]. Therefore, translating the ATI technique on linear probes could also provide significant clinical benefit in multiple imaging areas. 

This study is the first to evaluate the ability of a linear probe to successfully measure the attenuation in certified phantoms for liver tissue using a clinically approved system, the Aplio i800 ultrasound machine. The effects of different sizes and depths of regions of interests (ROIs), probe angles, handheld versus fixed probe and additional layer of skin and fat on to the phantoms were investigated.

## 2. Materials and Methods

### 2.1. Ultrasound

The study was completed using the ultrasound machine TUS-AI800 (Aplio i800, Canon Medical System Corporation, Otawara-shi, Japan). Attenuation imaging on liver tissue phantoms was completed with the commercially available Canon linear array i11LX3. For the purposes of this study, the linear probe was used in conjunction with the pre-set ATI settings installed on the Aplio i800 by Canon. The pre-set used was ATI-Gen, (attenuation imaging general), resulting in a frequency of 7 MHz, with a non-adjustable focal depth of 5.8 cm. The ATI-Gen is color coded and ranges from light blue to orange depending on the amount of received signal. While light blue stands for areas with enough signal to obtain accurate values (R^2^ > 0.9), orange-colored areas are not reflecting enough signal and should not be used for measurements. The R^2^ value of greater than 0.9 is recommended as per the Canon medical handbook. The R^2^ value is used as a coefficient of determination and calculated using Canon’s line profile of the average intensity within the placed ROI, and it can be used during a clinical measurement to avoid structures such as vessels and areas with lower signal due to scattering [28]. The measurements were taken regardless following protocol. This study uses proprietary technology in the form of linear probe-based attenuation imaging, which is currently not commercially available.

To ensure adequate contact between phantoms and the ultrasound probe, as per standard operating procedures with patients, ultrasound gel (UL-01, Skintact, Healthlife, Oldbury, UK) was used.

### 2.2. Liver Tissue Phantoms and Experimental Set Up

The phantom models (Model 039) were purchased from Computerized Imaging Reference System, Inc. (CIRS, Norfolk, VA USA). Certified attenuation values of each phantom are shown in Table 1.

**Figure 1:**
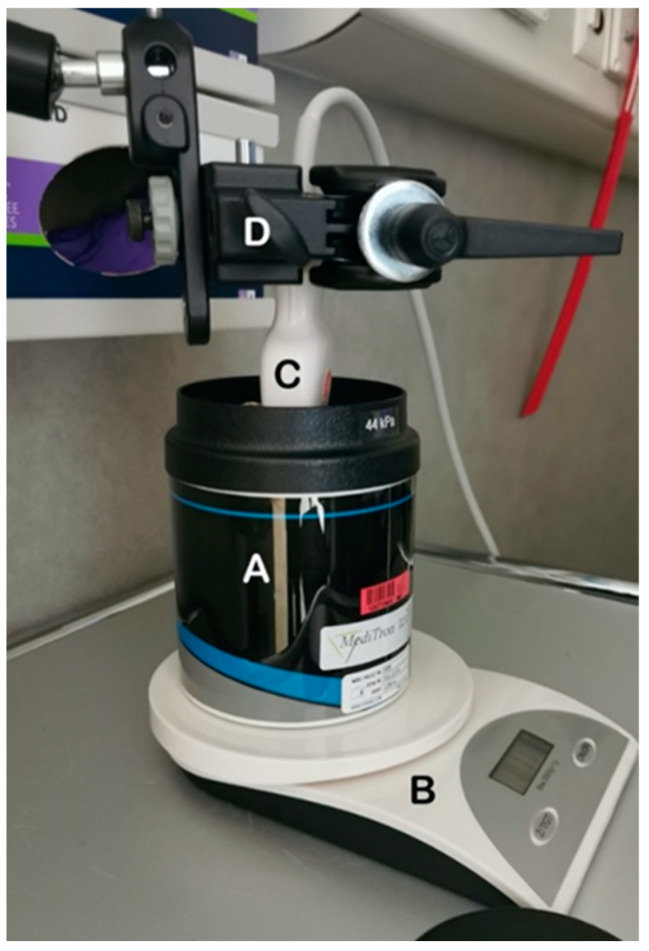
Image showing the setup used to record measurements. The phantom (A) was placed on a balance (B) to monitor for applied pressure changes between measurements. The ultrasound probe (C) was held in place by the mechanical arm (D).

A mechanical arm (Figure 1) was employed to ensure that the probe could be placed in the same position to repeat measurements. Prior to measurements commencing, the phantoms were placed on a scale, the scare was tared, and then the probe was placed in contact with the surface of the phantom using ultrasound gel and held in place with the mechanical arm. Weighing the phantom was performed to ensure no additional pressure was being applied between measurements with each phantom or change in technique with a change in mass of less than 100 g deemed acceptable. 

### 2.3. Experimental Protocol

**Figure 2:**
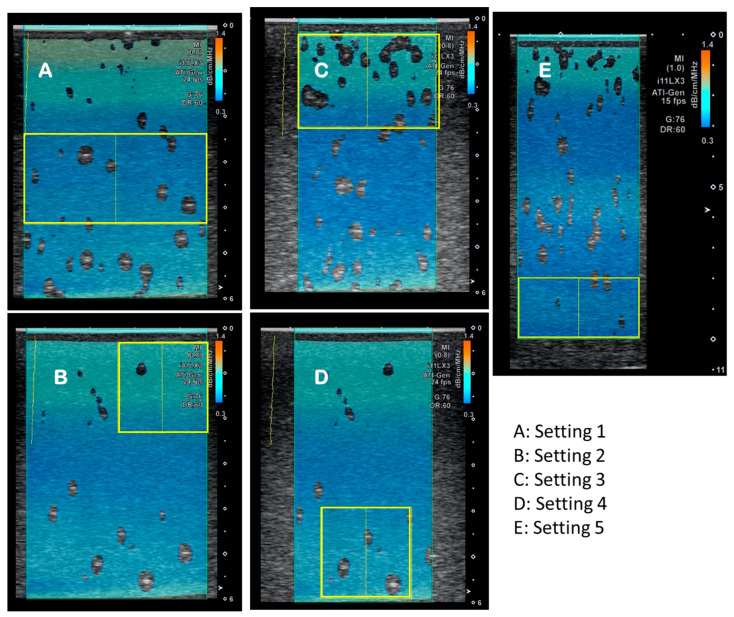
Example images showing the different settings **A**, **B**, **C**, **D** and **E** used with area of interest (AOI, blue) and region of interest (ROI, yellow box) with sizes as described in Table 2. The different measurement sizes, (**A**–**E**) were used to determine confounders for measurements.

The operator chose a 2D ATI-image as area of interest (AOI) and selected the region of interest (ROI) (Figure 2, Table 2). To ensure the effect of ROI size and AOI size played no significant role in measurement values, all measurements were taken with AOI, and ROIs are shown below. Methods A and B used the same size and depth for AOI but different size ROIs. Method C and D used the same AOI size and different ROI sizes. Method E used the same size AOI and ROIs as A but with the additional depth of 6–10 cm. The “centered” measuring sessions were completed using the linear probe in a vertical position to the phantom placed in the middle of the phantom and held by the mechanical set up (as described in Figure 1) at a 90° angle. For measurements using a fat layer and taken at an angle of 60°, the measured depths were corrected for the addition of a fat layer or the angle of the probe.

### 2.4. Measurements

**Figure 3:**
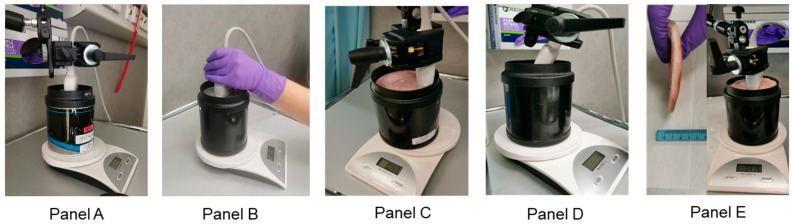
Images of the different configurations for measurements: Probe placement in the center (**A**), with a handheld technique (**B**), at the edge of the phantoms (**C**), at a different angle (60 degrees) to the phantom surface (**D**) and using a porcine fat layer (**E**) to mimic the effects of tissue on the phantoms (**left**) with this layer then placed on top of each phantom (**right**).

After the completion of initial centered measurements (Figure 3, Panel A), a further four different types of measurements were carried out to investigate additional variables leading to changes in measured attenuation. The same measurements as with the mechanical arm were repeated holding the probe in place by hand (Figure 3, Panel B) and at the edges of the phantom instead of centered (Figure 3, Panel C). The angle of the probe with regard to the phantom was examined (Figure 3, Panel D) with five measurement sessions, each examining the five different settings as with the central fixed arm measurements. The probe was fixed in a 60° angle to the surface of the phantom. Further measurements were completed using a fat layer of pork belly (approximately 1 cm thick) to mimic a tissue layer (Figure 3, Panel E, left) placed on top of the phantom (with ultrasound gel) to simulate the skin, fat and muscle layers in volunteers and patients (Figure 3, Panel E, right).

Repeat measurements of each type of measurement were made, with a complete removal of the probe and resetting up of the probe conducted between repeats. The number of measurements for each position were as follows, *N* = 10 for center measurements with mechanical arm, and *N* = 5 for all other positions. The measurements were conducted in sets for each position, with 5 repeats for each set, *N* = 10 for center and, *N* = 5 for each other position. After each initial set of measurements, including all repeats, the probe was removed and replaced in the same position to acquire a new set of measurements. Following a pilot study where the center position was measured and evaluated (*N* = 5), the complete study was repeated with *N* of 5 for each position.

### 2.5. Statistics

The statistical analysis was conducted in Python using the “bioinfokit” package (version number 3.11.2) for the two-way ANOVA and the “scipy” package for the *t*-tests. A two-way ANOVA for positioning of the probe and the depth of the measurements was conducted. A *t*-test was used to assess whether measured values differed significantly from the certified values. Two-sample *t*-tests were performed to compare the different positions to the “centered” measurements. A *p*-value of less than 0.05 was considered statistically significant; *p*-values of less than 0.05, 0.01 and 0.001 are presented in heatmaps.

## 3. Results

**Figure 4:**
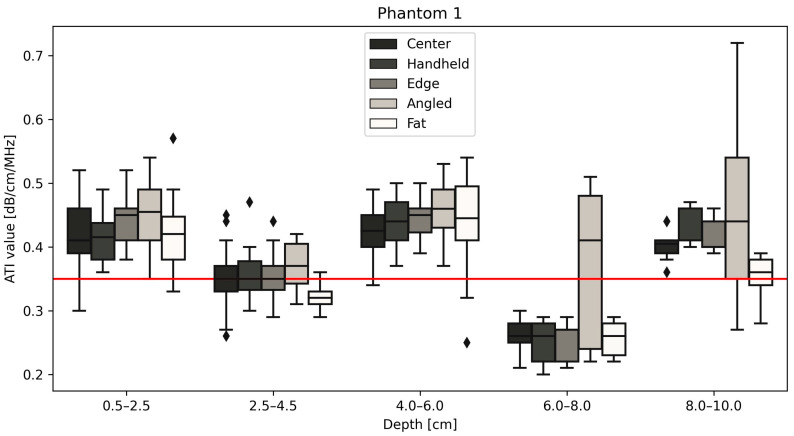
Attenuation values in (dB/cm/MHz) for five depths for phantom 1 at five positions. Measurements taken at depths lower than 6 cm are significantly different from the certified value (red line) provided by CIRS. The diamond shapes are outliers.

**Figure 5:**
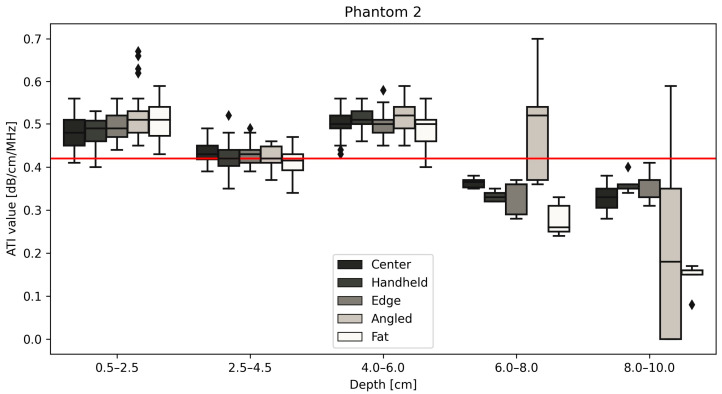
Attenuation values in (dB/cm/MHz) for five depths for phantom 2 at five positions. Measurements taken at depths lower than 6 cm are significantly different from the certified value (red line) provided by CIRS. The diamond shapes are outliers.

**Figure 6:**
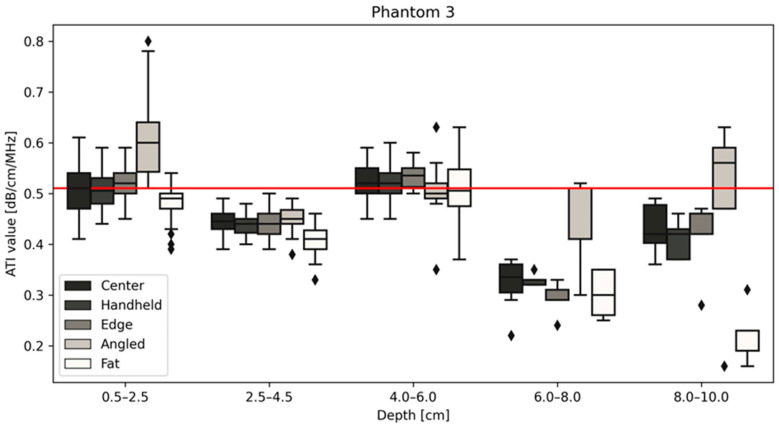
Attenuation values in (dB/cm/MHz) for five depths for phantom 3 at five positions. Measurements taken at depths lower than 6 cm are significantly different from the certified value (red line) provided by CIRS. The diamond shapes are outliers.

All measurements of the different positions are displayed for phantom 1 (Figure 4), 2 (Figure 5) and 3 (Figure 6) with the red line indicating the certified value of each phantom. Changing the size of the ROI or AOI had no significant effect on measurements taken, which is unsurprising given the homogeneity of the phantoms. This is unlikely to remain true when measuring in vivo, due to the heterogeneity of tissue, with the presence of naturally occurring scatterers and presence of large blood vessels. The ability to change the size of the ROI is an important one to be able to obtain accurate measurements without including such naturally occurring scatterers. The values closest to the certified values were taken at a depth of 2.5–4.5 cm for phantoms 1 (measured median = 0.35 vs. certified = 0.35 dB/cm/MHz, Figure 4) and 2 (measured median = 0.43 vs. certified = 0.42 dB/cm/MHz, Figure 5), while for phantom 3 (Figure 6), measurements at a depth of 0.5–2.5 cm showed values closest to the certified value (measured median = certified = 0.51 dB/cm/MHz). Examining the effects of depth on measurement values shows that for depths below 6 cm, the values can fluctuate greatly (Figure 4, Figure 5 and Figure 6, Table 3), specifically for measurements using a tilted probe (“angled”). The values differ significantly from the certified values.

Figure 4 shows the results for phantom 1 at different depths and under different measurement conditions. The red line on the figure denotes the certified value of the phantom, 0.35 dB/cm/MHz. The measurements taken for the center, handheld, edge, angled and with the fat layer included are shown to be most reliable at a depth of 2.5–4.5 cm, across each measurement type, with the median values closest in value to the certified values of the phantom. This is similar to the results shown below in Figure 5. 

Figure 5 shows the measurements obtained using the probe for a phantom with a certified value of 0.42 dB/cm/MHz. The results follow those of the results for phantom 1 (Figure 4) in that values taken from 2.5–4.5 cm deep have the greatest reproducibility and are closest in value to that of the phantom. 

Figure 6 demonstrates a change in the trend between phantoms 1 and 2, in that phantom 3 measurement values at a depth of 4.0–6.0 cm are closest to the certified values for the phantom, 0.51 dB/cm/MHz. The common change in measurement values post 6.0 cm deep remains true for all three phantoms regardless of measurement type, as can be seen below in Table 3.

**Figure 7:**
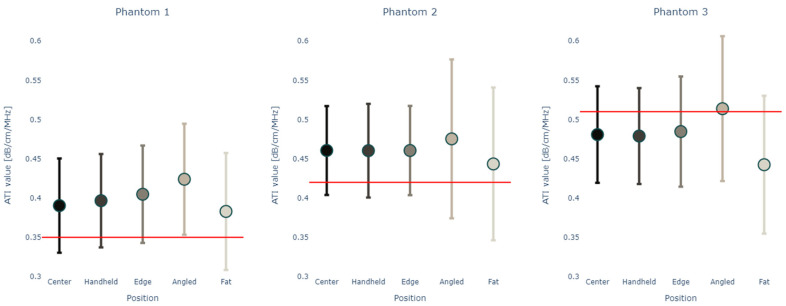
Mean and standard deviation of attenuation values in (dB/cm/MHz) for depths 0.5–6 cm for each measurement position versus the certified value (red line) in phantoms 1–3, respectively.

Due to this significant difference between the measured values at depths lower than 6 cm and the certified values for all phantoms, values taken at positions 6–8 cm and 8–10 cm were excluded from further statistical analysis. The two-way ANOVA showed that depth had a significant impact on attenuation values with a *p*-value < 0.0001, as did the position within the phantom with *p* = 0.0025. Further analysis examines the relationships between confounders and attenuation at depths from 0.5–6 cm, where the standard deviation for each measurement type is small and measurements within each phantom are consistent with each other (Figure 7, Table 4). 

**Figure 8:**
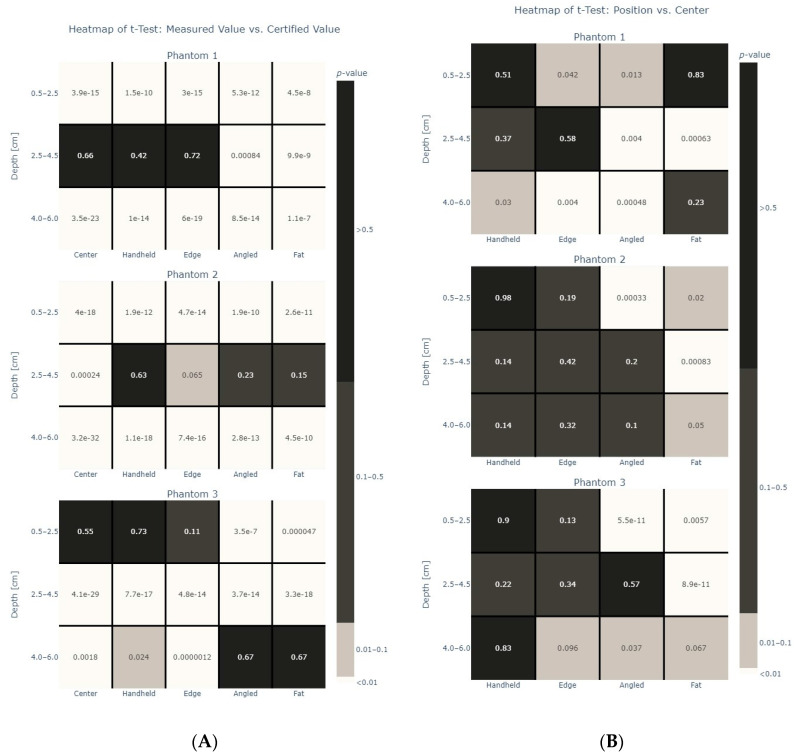
(**A**) Heatmap for each phantom, showing the relationship between measured values and certified values for each phantom. Values here represent *p*-values. (**B**) Heatmap for each phantom, showing the relationship between centered measured values and values for each other position in each phantom. *p*-values greater than 0.5 are shown in black, *p*-values between 0.1–0.5 are shown in gray, *p*-values between 0.01–0.1 are shown in light gray and *p*-values less than 0.01 are shown in white.

Figure 8A shows the results from the *t*-tests when comparing the measurement type versus the certified value for each phantom with Figure 8B showing the heatmaps for the same *t*-tests when comparing the centered measurements with measurements in other positions. Again, it can be seen that measurements at a depth of 2.5–4.5 cm for phantoms 1 (*p*-value > 0.5 for “center”, “handheld”, “edge”) and 2 (*p*-value > 0.5 for “handheld”), respectively, at a depth of 0.5–2.5 cm for phantom 3 (*p*-value > 0.5 for “center”, “handheld”) are closest to the certified expected values for each phantom. Center and handheld measurements were having very similar results while the tilting of the probe and the addition of a fat layer changed the values more.

## 4. Discussion

Results show the feasibility of ATI measurements using a linear probe in phantoms. Measuring at different depths and positions has a significant impact on the values. The results show repeatability of measurement values within the range of 0.5–6.0 cm, therefore fulfilling a requirement to be implemented in clinical routine. Positions with the ultrasound probe perpendicular to the surface of the phantoms such as centered, handheld and edge were closer to the certified values than measurements taken with a 60-degree angle to the surface. Adding a fat layer on top of the surface of the phantom changes the depth and therefore has a significant impact on the measurements.

Ultrasound attenuation imaging (ATI) using convex ultrasound probes has been explored to diagnose, monitor and predict disease progression in patients. Specifically, attenuation measurements on convex probes have been used to monitor hepatic steatosis in patients with chronic liver disease [29] as well as for evaluating fatty liver in children [30]. In these investigations, depth is also explored, with depths up to 9 cm being measured. Clinically, a linear probe, operating between 5 and 12 MHz, and thus a higher frequency than the standard convex probe, does not return the same image quality at depths exceeding 7 cm [31]. 

Our study confirmed where the limitations lie with unreliable measurements at depths greater than 6 cm deep using a linear probe for attenuation imaging. The echo intensity of ultrasound is depth- as well as probe angle-dependent [32,33], and previous studies have shown that shear wave elastography on the linear probe seems to be significantly influenced by depth as well as chosen ROIs [34]. Jesper et al. presented similar findings in a study about ATI in phantoms using a convex probe, where the values significantly decreased with lower depth (further distances from the probe to the chosen ROI) [10]. The same effect of depth, ROI size and angle of the probe has been shown in this study on ATI measurements using a linear probe.

As well as the prospect of using ultrasound attenuation coefficients for predicting liver disease and progression, ATI has been investigated in different tissues using convex probes. Areas of investigation have included the link between attenuation coefficients and the type of tissue that the ultrasound is passing through, e.g., normal, benign or malignant breast tissue [35,36]. This is one area that warrants new investigation with the latest methods, as progression in this area has stalled in recent years. Previously ultrasound attenuation has been one metric by which researchers have tried to formulate new methods for diagnosing and monitoring patients.

Using self-constructed phantoms, Nam et al. have previously examined a linear array on four systems [37], two of whom have been clinically approved, the Siemens Acusion S2000 and Zonare Z. In both studies, radiofrequency data were recorded and fitted to mathematical models, giving estimates based on a reference phantom. Our study presents the first to investigate confounders and the first using reference phantoms on a linear array. Real-time attenuation measurements were performed without usage of fitting to models or estimates.

One keynote from this study is the comparison between “techniques”. A mechanical arm was used to hold the probe in place for four out of the five measurement types: center, edge, angled and “fat” measurements. These results align well with the results for the handheld measurements which were completed without the use of a mechanical arm. This would indicate a robust method and that the system is able to produce repeatable values. The effect of changing the measurement position can be seen in Figure 8. Specifically, a significant effect can be seen when changing the angle of the probe or by adding a “fat” layer. A “fat” layer was chosen to represent clinical conditions that the probe may be used in. The phantoms are liver phantoms, and they accurately represent the acoustic properties of liver tissue; however, clinically for the ultrasound beam to penetrate the liver, there are layers of skin and fat that must also be penetrated. For a clinically beneficial protocol to be established, the impact on the measurements of a thick fat layer on top of the liver tissue was explored. As seen in the results, the fat layer did indeed influence the measurement values. The effect of changing the angle or adding a “fat” layer is unsurprising given that these factors are well known to have an effect on attenuation. The major considerations for attenuation in ultrasound imaging include absorption, scattering, reflection, diffraction, frequency and interference [38]. 

Each phantom displayed a change in attenuation values with a change in angle. The reason for the changes in values may be attributed to some of the factors discussed above. For example, the phantoms contain scatterers to simulate some of the biological properties of a liver when ultrasound imaging. A change in the angle at which the ultrasound beam interacts with these scatterers may result in a change in attenuation values. However, there is likely to be a greater effect from off-axis reflection of the incident beam at the surface of the phantom, resulting in a loss of signal being returned to the probe and therefore greater attenuation. Changing the angle of the measurement also influenced the measurement values for each phantom in the 6–8 and 8–10 cm measurement range, respectively. In phantoms 1 and 3 specifically, a large drop and then increase in attenuation can be seen occurring at these two depths. This is likely caused by backscatter, which changes when the angle is changed, meaning an elimination of those affects at those depths. Finally, by changing the angle, sufficient coupling of the probe to the phantom is required to ensure sufficient contact. 

A similar hypothesis can be drawn for the significant effect that the fat layer had on attenuation values. The layer adds a new tissue property with a different effect on the beam. The layer introduces an additional absorption with new scattering, reflecting and diffracting properties before the beam reaches the tissue phantom, leading to a change in values. The addition of a fat layer added a realistic attenuator not otherwise present with use of the phantoms. Fat is known to attenuate ultrasound, and this is an important consideration when using ultrasound at a higher frequency. The standard measurements for liver attenuation are currently completed using a convex probe with center frequencies ranging from 3 to 5 MHz; however, as the linear probe used in this study had a higher center frequency of 7 MHz, the use of a fat layer to ensure clinical relevance was used. 

A final consideration is of the frequency of the probe. With an increased frequency, there is an increase in attenuation [38]. This has been long known and understood with ultrasound imaging: the trade-off between spatial resolution and penetrating depth. However, it is important to take into consideration for the results presented here. The use of linear probes, clinically, are used for superficial structures due to their high resolution; however, this comes at a cost of penetration depth. This is readily apparent in this study, as with the increase in the depth, the attenuation values differ significantly, with higher frequencies attenuating more readily due to absorption and scattering. The effects of depth can be attributed to the frequency of the probe, but also the elevation focus of the machine must be considered as a reason for differences in values of 0.5–2.5 cm and 2.5–4.5 cm. 

The effect of depth was also apparent in this study. As can be seen from the results, there is a change in attenuation between 6–8 cm and 8–10 cm that may be unexpected. This is likely due to backscatter from the plastic casing of the phantoms. The phantoms have a limited depth of 12 cm, which is close enough to the measurement area of 8–10 cm to cause as variation in measurement values. This could be mitigated against in future studies by an acoustic absorber being incorporated; however, at the time of the study, this was not possible. 

The main limitation of this study is that the measurements have only been performed on phantoms, while real liver tissue may show slightly different characteristics. Below 6 cm deep into the phantoms, regardless of measurement type, the reproducibility and precision of the method drops. This limitation is one that is expected to occur when using a high-frequency linear probe. Nonetheless, this must be taken into consideration when exploring areas for further research with the disease models to be explored examined closely as to whether linear attenuation imaging is the method that will work best and provide the most accurate results. A final limitation is not knowing the phantoms that were used by Canon to calibrate their ultrasound machines and being able to compare this phantom directly with linear attenuation values. A further limitation includes the attenuation values of the phantoms. With a range of 0.35–0.51 dB/cm/MHz, there is not a phantom that has an attenuation value that would fall in the expected range for steatosis, >0.53 dB/cm/MHz [39]. 

Using a convex probe, the novel ATI metric has been investigated in the differentiation of different tissue types (e.g., normal, benign or malignant breast tissue) [35,36,40] as well as the diagnosing and monitoring patients [41,42,43]. Clinical fields that might particularly profit from a linear probe due to the higher frequency include musculoskeletal (e.g., sarcopenia), breast (breast density and cancer assessment) and thyroid imaging.

## 5. Conclusions

A new method of measuring ultrasound attenuation in standardized liver tissue phantoms was established and examined in a Canon Aplio i800 using a linear probe. Measurement values were compared to the certified values of the tissue phantoms and have been shown to be in line with those. Positioning and tilting of the ultrasound probe as well as depth of measurement was found to influence the attenuation values and provides important information when moving forward with future volunteer studies. Attenuation imaging using a linear probe seems to be most accurate in depths to a maximum of 6 cm.

## Figures and Tables

**Table 1:** Certified CIRS values for each attenuation phantom.

Phantom	Certified Attenuation Value (dB/cm/MHz)
1	0.35
2	0.42
3	0.51

**Table 2:** Different settings for region of interest (ROI) size choice.

Setting	ROI	Depth
A	4 cm × 2 cm = 8 cm^2^	0.5–2.5 cm, 2.5–4.5 cm, 4–6 cm
B	2 cm × 2 cm = 4 cm^2^	0.5–2.5 cm, 2.5–4.5 cm, 4–6 cm
C	3 cm × 2 cm = 6 cm^2^	0.5–2.5 cm, 2.5–4.5 cm, 4–6 cm
D	2 cm × 2 cm = 4 cm^2^	0.5–2.5 cm, 2.5–4.5 cm, 4–6 cm
E	4 cm × 2 cm = 8 cm^2^	0.5–2.5 cm, 2.5–4.5 cm, 4–6 cm, 6–8 cm, 8–10 cm

**Table 3:** Median attenuation values for each position and each phantom measurement are displayed with changing depths.

**Phantom 1** **Median ATI values**		**Depth**				
		**0.5–2.5 cm**	**2.5–4.5 cm**	**4–6 cm**	**6–8 cm**	**8–10 cm**
Position	Center	0.410	0.350	0.425	0.260	0.405
	Handheld	0.415	0.350	0.440	0.260	0.410
	Edge	0.450	0.350	0.450	0.270	0.400
	Angled	0.455	0.370	0.460	0.410	0.440
	Fat	0.420	0.320	0.445	0.260	0.360
**Phantom 2** **Median ATI values**		**Depth**				
		**0.5–2.5 cm**	**2.5–4.5 cm**	**4–6 cm**	**6–8 cm**	**8–10 cm**
Position	Center	0.480	0.430	0.500	0.365	0.330
	Handheld	0.490	0.420	0.510	0.330	0.350
	Edge	0.490	0.430	0.500	0.360	0.370
	Angled	0.510	0.420	0.520	0.520	0.180
	Fat	0.510	0.415	0.500	0.260	0.160
**Phantom 3** **Median ATI values**		**Depth**				
		**0.5–2.5 cm**	**2.5–4.5 cm**	**4–6 cm**	**6–8 cm**	**8–10 cm**
Position	Center	0.510	0.445	0.520	0.335	0.420
	Handheld	0.505	0.440	0.520	0.330	0.420
	Edge	0.520	0.440	0.535	0.290	0.460
	Angled	0.600	0.450	0.500	0.410	0.560
	Fat	0.490	0.410	0.505	0.300	0.190

**Table 4:** Mean attenuation values (dB/cm/MHz) and standard deviation for each position and each phantom measurement at depths from 0.5 to 6 cm are displayed with the certified values.

	Phantom 1			Phantom 2			Phantom 3		
	Mean	Standard Deviation	Certified Value	Mean	Standard Deviation	Certified Value	Mean	Standard Deviation	Certified Value
Center	0.397	0.054	0.35	0.473	0.043	0.42	0.492	0.048	0.51
Handheld	0.403	0.050		0.473	0.048		0.491	0.049	
Edge	0.413	0.053		0.473	0.042		0.499	0.051	
Angled	0.425	0.056		0.488	0.062		0.520	0.084	
Fat	0.392	0.071		0.469	0.056		0.463	0.062	

## Data Availability

All research data are available upon request to corresponding author.
